# The Development Of *Drosophila Melanogaster* under Different Duration Space Flight and Subsequent Adaptation to Earth Gravity

**DOI:** 10.1371/journal.pone.0166885

**Published:** 2016-11-18

**Authors:** Irina V. Ogneva, Stepan N. Belyakin, Svetlana V. Sarantseva

**Affiliations:** 1 Cell Biophysics Group, State Scientific Center of Russian Federation Institute of Biomedical Problems of the Russian Academy of Sciences, Moscow, Russia; 2 I. M. Sechenov First Moscow State Medical University, Moscow, Russia; 3 Institute of Molecular and Cellular Biology SB RAS, Novosibirsk, Russia; 4 B. P. Konstantinov Petersburg Nuclear Physics Institute National Research Centre "Kurchatov Institute", Gatchina, Russia; Charles P. Darby Children's Research Institute, UNITED STATES

## Abstract

In prospective human exploration of outer space, the need to preserve a species over several generations under changed gravity conditions may arise. This paper demonstrates our results in the creation of the third generation of fruit fly *Drosophila melanogaster* (third-stage larvae) during the 44.5-day space flight (Foton-M4 satellite (2014, Russia)), then the fourth generation on Earth and the fifth generation again in conditions of the 12-day space flight (2014, in the Russian Segment of the ISS). The species preserves fertility despite a number of changes in the level of expression and content of cytoskeletal proteins, which are the key components of the cleavage spindle and the contractile ring of cells. The results of transcriptome screening and space analysis of cytoskeletal proteins show that the exposure to weightless conditions leads to the increased transcription of metabolic genes, cuticle components and the decreased transcription of genes involved in morphogenesis, cell differentiation, cytoskeletal organization and genes associated with the plasma membrane. “Subsequent” exposure to the microgravity for 12 days resulted in an even more significant increase/decrease in the transcription of the same genes. On the contrary, the transition from the microgravity conditions to the gravity of Earth leads to the increased transcription of genes whose products are involved in the morphogenesis, cytoskeletal organization, motility of cells and transcription regulation, and to the decreased transcription of cuticle genes and proteolytic processes.

## Introduction

The appearance of life on Earth and the evolution of all living organisms occurred under the influence of external physical fields: the gravitational field and the electromagnetic field. The most constant external physical field is certainly the gravitational field. The formation of a living cell under these physical conditions means that its structural and functional characteristics must enable it to exist under the influence of these physical fields.

A number of various evolutionary solutions were developed for the problem of preserving the organism’s own mass in the gravitational field of Earth [1,2,3]. The changed gravity leads to changes in various cellular processes and structures, such as proliferation, signalling pathways, and cytoskeletal organization [4,5]. However, the mechanism for the primary reception of the external mechanical field is still unclear.

There is no need to adapt to changes in gravity on Earth; the usual model experiments change the orientation of the study object in the gravitational field but do not prevent the gravity influence. Therefore, the most interesting data are the results obtained in actual weightless conditions.

*Drosophila* was one of the first organisms that were used to determine the gravity influence on the early development of embryos [6]. The results of early experiments in space showed the normal development of *Drosophila* in the space conditions [7]. However, the results obtained during the last successful 7-day flight of Challenger Shuttle (November 1985, USA) showed that the oogenesis and embryonic development of *Drosophila* change in the absence of gravity [8]. In the space flight conditions the following was observed: increase in generation of egg cells and increase of their size, significant reduction in the number of newly hatched larvae, morphological changes in the head and thoracic segments of the imago. The group exposed to 1g centrifugation in space showed no such changes [8]. However, further experiments conducted by the same group of authors [9] using the equipment that allows to improve the oxygenation of samples demonstrated the normal quantitative and quality development of *Drosophila* in the microgravity conditions, at least if the mature larvae and adult flies of *Drosophila* are studied.

In general, *Drosophila* is an optimal model for a screening analysis of the cellular changes resulting from the changed external mechanical field, based on its short life cycle and relatively small genome. These features make it possible to identify new approaches to solve the fundamental problem of cell mechanoreception. Furthermore, the possibility of analysing the influence of gravity on the development of a complex multicellular organism may be of interest with regard to the evolutionary aspect.

Given this information, the main purpose of our research was to create a third generation of fruit fly larvae under space flight conditions (in the Foton-M4 satellite) and perform a screening analysis of the changed expression of different genes and a target analysis of the genes that encode cytoskeletal proteins in the third generation immediately after landing and to determine the changes arising during the adaptation to the gravity of Earth and after the subsequent return to the planetary orbit (in the Russian Segment of the International Space Station).

## Materials and Methods

### Experimental design

The research material was the third-stage larvae of *Drosophila melanogaster* Canton S line obtained after the 44.5-day space flight of the Foton-M4 satellite (flight dates: 19.07.14, 0:50 Moscow time, Baikonur, Kazakhstan– 01.09.14, 13:20 Moscow time, Orenburg Region, Russia) and the 12-day space flight in the Russian Segment of the International Space Station (29.10.14, 10:06 Moscow time, Baikonur, Kazakhstan– 10.11.14, 06:58 Moscow time, an area to the north of Arkalyk, Kazakhstan).

The research objects were placed in the Foton-M4 satellite in 50-ml plastic Falcon-type tubes in three BB-2 blocks of the BB-1F scientific equipment and exposed to passive oxygenation using the air medium of the satellite. Thirty tubes with air-permeable stoppers were placed into each BB-2 block. The tubes contained the firm standard (for breeding *Drosophila*) nutrient medium with the following composition: water– 1,000 ml, agar-agar– 7 g, sugar– 40 g, semolina– 40 g, baker’s yeast– 25 g, and propionic acid– 10 ml.

**Fig 1:**
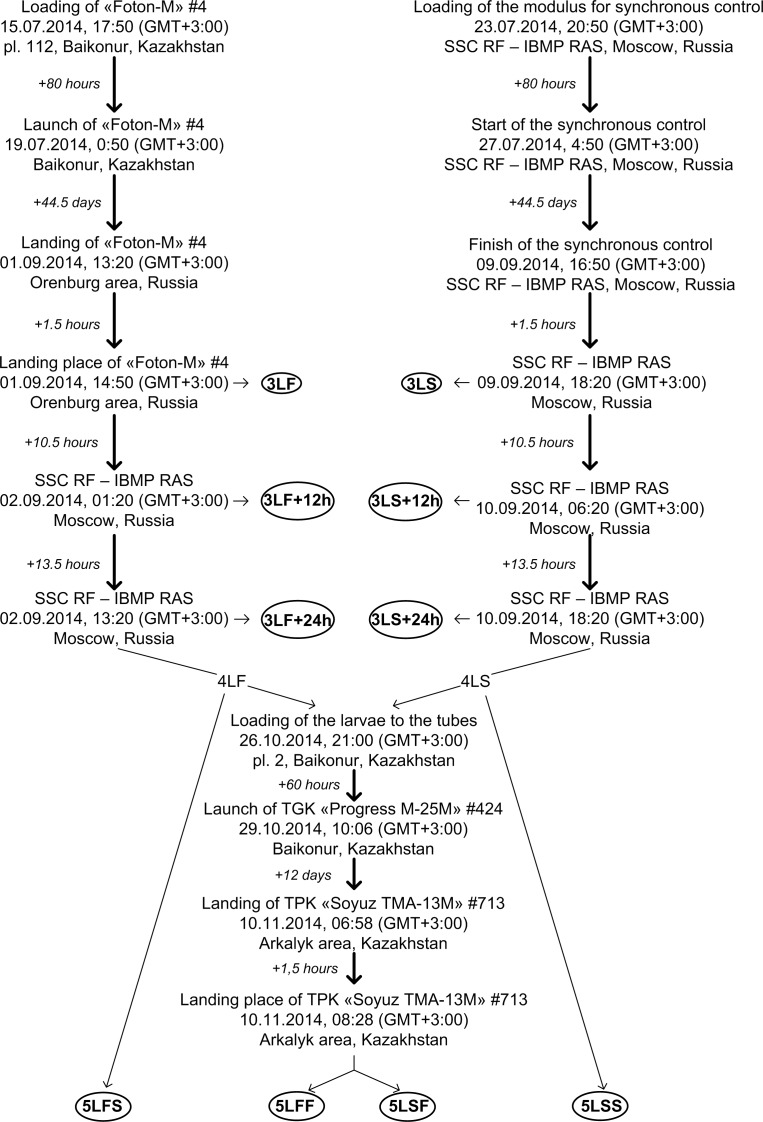
Design and cyclogram of the experiment. The experimental groups are highlighted by the circles.

Eighty hours before the launch of the satellite, the imago of *Drosophila melanogaster*, Canton S line (3–5 pairs of males and females), aged 2 days, were placed in the tubes that were subsequently placed in the BB-1F scientific equipment (Fig 1). All procedures were conducted at room temperature (24–26°C).

The temperature and gas composition of the medium were controlled during the placement of the equipment on the launch pad, and during the satellite’s flight, the medium status in the satellite was monitored. During the flight, the average temperature was 18.1±2.4°C, the unit pressure was 100529 Pa, the O_2_ content was 135±11 mm Hg, the CO_2_ content was 2.22±0.17 mm Hg, and the relative humidity was 40±7%. The synchronous ground monitoring study was conducted under the same temperature conditions and air medium composition similar to the values measured during the satellite’s flight. Biological object preparation and loading procedures, as well as the distribution of the biomaterial for research after the end of the experiment were similar to the procedures applied before the launch of the satellite.

The distribution and recording of the biomaterial after the satellite landed were conducted as described below. The biomaterial (third-stage larvae) from the first BB-2 block was recorded 1.5 hours after the satellite landed (3LF group–the 3^rd^ generation larvae during the flight), 12 hours after the satellite landed from the second BB-2 block (3LF+12h group,), and 24 hours after the satellite landed from the third BB-2 block (3LF+24h group). Some of the larvae remained in the tubes to create adult flies for re-exposure in space.

We used early third instar larvae for our experiments. After receiving the containers at the landing place, several larvae were dissected to determine the presence of food in their gut. We estimated size of larvae, which had food in their gut and, next, collected larvae with the same size due to limited time at the landing place.

Thus, the control groups were formed from the material of synchronous control: 3LS group (third-stage larvae recorded 1.5 hours after the end of the experiment), 3LS+12h group (larvae recorded 12 hours after the end of the experiment), and 3LS+24h group (larvae recorded 24 hours after the end of the experiment).

Following the landing of the Foton-M4 satellite, the unrecorded 3^rd^ generation larvae were used to create the imago, which subsequently generated the 4^th^ generation. Similarly, the 3^rd^ generation larvae obtained during the synchronous control also generated the 4^th^ generation. The 4^th^ generation larvae of the third stage of development (5 pairs of males and females) were sent to the Russian Segment of the ISS in Progress M-25M424 spacecraft. Twelve days after landing with the ISS 40/41 crew in Soyuz TMA-13M713 spacecraft, the living 4^th^ generation imago and the 5^th^ generation larvae were created. The material was recorded on the landing site 1.5 hours after the landing of the spacecraft.

After the 12-day space flight, the following study groups were formed: 5LFF–the 5^th^ generation larvae of the line from which the 3^rd^ generation larvae were created during the flight of the Foton-M4 satellite, and 5LSF–the 5^th^ generation larvae of the line from which the 3^rd^ generation larvae were created as a result of the synchronous experiment during the flight of the Foton-M4 satellite. Due to the limited amount of biomaterial in the 5LSF and 5LFF groups (the weight of useful load delivery to the ISS is limited), we had no opportunity to analyse the adaptation effects 12 and 24 hours after the end of the 12-day flight.

The synchronous ground experiment conducted during this 12-day flight (under the same temperature conditions and air medium composition similar to the values measured during the space flight at the ISS board) also allowed us to create the 5^th^ generation larvae and form the following groups: 5LFS–the 5^th^ generation larvae of the line from which the 3^rd^ generation larvae were created during the flight of the Foton-M4 satellite, and 5LSS–the 5^th^ generation larvae of the line from which the 3^rd^ generation larvae were created as a result of the synchronous experiment during the flight of the Foton-M4 satellite.

### Transcriptome-wide analysis using the NGS method

The total RNA was isolated from the larvae of all study groups using TRIzol reagent (Invitrogen, USA) according to the manufacturer’s instructions. For the subsequent transcriptome analysis, equal amounts of RNA from each sample were selected and the cDNA libraries were prepared using Illumina TruSeq RNA Library Preparation Kit v2 (Illumina, USA), according to the manufacturer’s instructions. The resulting fragment libraries were sequenced using Illumina MiSeq (Illumina, USA). The reading length was 250 bps at each end. Approximately 10-14 million paired 250 bp readings were obtained for each sample. The data obtained after sequencing (fully data are presented in GEO database, number GSE88866, https://www.ncbi.nlm.nih.gov/geo/query/acc.cgi?acc=GSE88866) were analysed using Galaxy membrane (www.usegalaxy.org). The intersection search and the comparison of direct and return readings, as well as the alignment relative to the reference genome were performed using the tophat2 application, and the identification of transcripts was performed using Cufflinks [10]. The RPKM method (reads per kb per million reads) was used to determine the mRNA content of each gene [11]. The absolute log2ratio ≥1 was used as the limit to assess the differential gene expression in different comparison groups. The annotation of differentially expressed genes (DEG) was performed using the DAVID Gene Ontology [12,13] program (FDR<0.001), and the subsequent clustering was performed using the REVIGO package [14]. The genes were compared in pairs in groups, whereas the genes with increased or decreased expression were annotated separately. The information about the participation of these lists of genes in the biological processes under study, the localization of their products in the cell, or their molecular functions and involvement in signalling pathways were obtained.

### Determination of mRNA content using the quantitative PCR method

Due to the limited amount of biomaterial obtained after the experiments conducted in the real space flight conditions we did not have possibility to do NGS analysis for biological repetition. Moreover, we limited significance of changes in the NGS data by log2ratio ≥1 to assess the differential gene expression. So, we decided to verify NGS data by assessing the mRNA content of a number of cytoskeletal genes. By the other hand, we estimated genes, whose expression level was significantly changed, but is less than twice.

The total RNA was isolated from frozen tissues of the third-stage larvae using the RNeasy Micro Kit (Qiagen, Germany) according to the manufacturer’s instructions, to perform the target analysis of the mRNA content of genes that encode a number of cytoskeletal and metabolic proteins. Reverse transcription was performed using d(T)_15_, and 500 ng of RNA was used as an inoculum. Real-time PCR was performed using the primers selected via the Primer3Plus program (Table 1) to assess the expression levels of the studied genes. The 2(-Delta DeltaC(T)) method [15] was used to determine the fold change.

**Table 1:** Primer sequences and product sizes.

Gene	Primer sequence, forward/reverse (5'…3')	Product size, bp
*Act57B* (actin 57B, beta-actin)	*ccccatccacttgttaatcg/ tcggggaagttgttaggttc*	115
*Act87E* (actin 87E, beta-actin)	*aggaaccgcgattgtaacag/ tcttgtgtctcctcaactcctc*	96
*Act5C* (actin 5C, gamma-actin)	*gatcgggatggtcttgattc/ gtggttccgctcttttcatc*	149
*Arpc3*(actin-related protein 2/3 complex, subunit 3)	*cccaataaatggtggacctg/ acccgtcgtagaagcaaaac*	119
*Tmod* (tropomodulin)	*gacaaccaatccaaccaacc/ ctgacgtccaattcatgtcg*	70
*Svil* (supervillin)	*atgagggtggatcagctttg/ ttgaagcactggagttgcac*	120
*Fim* (fimbrin)	*agacctaccgcaattggatg/ agttgacaatacccggcttg*	126
*Actn* (alpha-actinin)	*acaagccgaacattgaggag/ gcgtttccatcgtgtagttg*	96
*Betatub85D* (beta-tubulin 2B)	*gtggcggcgatgaataatag/ atgctaaggcccaaagatcc*	119
*Msps* (mini spindles, CKAP 5)	*aaataacccccgaggaattg/ cttatttcgcccagaagctg*	127
*T-cp1* (T-complex 1-like)	*caagatcattggtgctgacg/ cacggggatctgtgattttc*	83
*Cct5* (T-complex chaperonin 5)	*ggagtgcaaaaactccaagg/ caccaccgtaaacaatacgc*	143
*T-cp1eta* (chaperonin containing Tcp-1, subunit 7—eta)	*atcgtgctcctcaaagaagg/ caggcattgatgttggacac*	71
*Cyt-c* (cytochrome c)	*tgctggtgatgttgagaagg/ agattgggtccaaccttgtg*	99
*Gapdh*(glyceraldehyde 3-phosphate dehydrogenase)	*aaagcggcagtcgtaatagc/ tcttcgacatggctgagttc*	80

### Determination of the protein content using the gel electrophoresis method followed by immunoblotting

Some of larvae were frozen in liquid nitrogen to determine the content of a number of cytoskeletal proteins. Tissue extracts were used to prepare the cytoplasmic and membrane fractions of the proteins [16,17]. Denaturing electrophoresis on polyacrylamide gels was performed using the Laemmli method and a Bio-Rad system (USA). Relying on the measured concentration, an equal amount of protein was placed into each well, separated by electrophoresis, and transferred to the nitrocellulose membrane [18]. Specific primary monoclonal antibodies were used at the dilutions recommended by the manufacturers to determine the levels of each protein: rabbit antibodies against total actin (Santa Cruz Biotechnology, Inc., USA) diluted 1:100, mouse antibodies against beta-actin antibodies (Abcam, UK) diluted 1:1,000, rat antibodies against actinin (Abcam, UK) at a concentration of 1 μg/ml, mouse antibodies against beta-tubulin (Santa Cruz Biotechnology, Inc., USA) diluted 1:100, and mouse antibodies against acetylated tubulin (Santa Cruz Biotechnology, Inc., USA) diluted 1:100. Biotinylated goat antibodies were used as the secondary antibodies to detect rabbit IgG (Jackson ImmunoResearch Lab, Inc., USA.) at a dilution of 1:10,000, to detect mouse IgG (Sigma, Germany) at a dilution of 1:20,000, and to detect rat IgG (Sigma, Germany) at a dilution of 1:20,000. Afterwards, all of the membranes were treated with a streptavidin solution conjugated with horseradish peroxidase (Sigma, Germany) diluted 1:8,000. Protein bands were detected using 3,3’-diaminobenzidine (Merck, USA). The ImageJ program was used to analyse the obtained data.

### Statistical analysis

The results obtained from the determination of the mRNA and protein contents were statistically analysed using ANOVA and the post hoc t-test, and a significance level of p<0.05 was applied to assess the reliability of differences between the groups. The data are provided in the form of M±SE, where M represents the arithmetic mean and SE represents the standard error of the mean.

## Results

### Effects of space flight factors on transcriptome

The expression analysis of the obtained samples was performed using the RNA-seq method based on the Illumina MiSeq platform. FPKM values were calculated using cufflinks^10^ software, with the genomic annotation of BDGP R5/dm3 (genome-euro.ucsc.edu). All data are presented in GEO database (GSE88866). The genes were considered differentially expressed if the expression was changed by at least two-fold compared to the corresponding control level.

**Fig 2:**
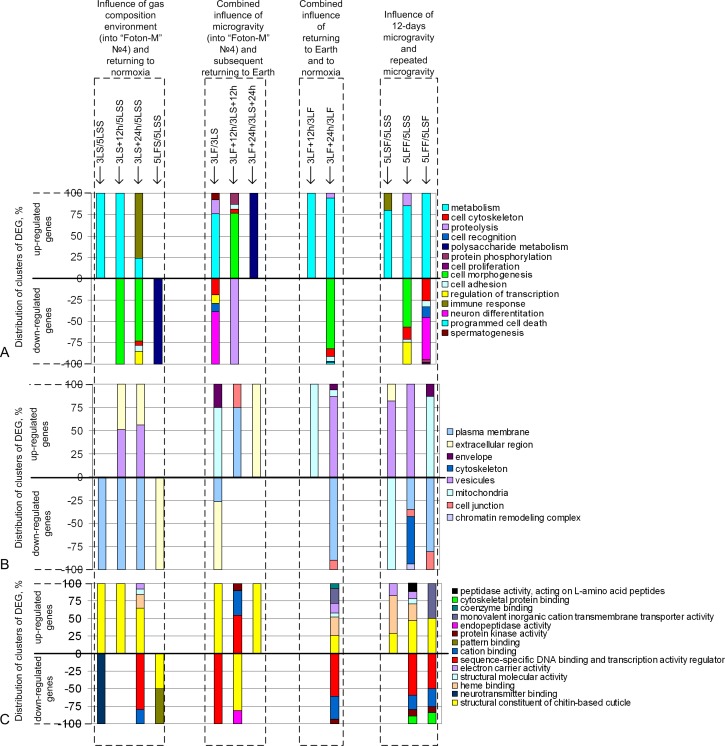
Distribution of differentially expressed genes among pairwise comparisons of the study groups. (A) DEG distribution according to the biological processes. (B) DEG distribution according to the cellular compartments. (C) DEG distribution according to the molecular functions.

The influence of the gas composition of the medium in the Foton-M4 satellite was assessed by comparing the synchronous control groups during the satellite’s flight and 5LSS group (first dotted rectangle in Fig 2A, 2B and 2C). Among the up-regulated genes (their numbers were 3LS group vs 5LSS group– 1132, 3LS+12h vs 5LSS– 954, 3LS+24h vs 5LSS– 860, and 5LFS vs 5LSS– 588), the dominant genes encoded proteins involved in oxidation-reduction processes and cuticle formation and were localized in vesicles and the extracellular space. The products of the down-regulated genes (their numbers were 3LS group vs 5LSS group– 1246, 3LS+12h vs 5LSS– 1276, 3LS+24h vs 5LSS– 2728, and 5LFS vs 5LSS– 1063) were involved in morphogenesis, cytoskeletal organization or were associated with the plasma membrane.

Immediately after the return to Earth (3LF vs 3LS, second dotted rectangle in Fig 2A, 2B and 2C) the transcription of metabolic genes and the genes involved in the cuticle formation was increased (1175 up-regulated genes), and the transcription of genes whose products are involved in neuronal differentiation, cytoskeletal organization was decreased (2666 down-regulated genes). Twelve hours after exposure to the gravity of Earth (3LF+12h vs 3LS+12h), the clusters of up-regulated genes (1192 genes) formed the genes whose products are involved in morphogenesis, transcriptional regulation and are associated with the plasma membrane. The clusters of genes with decreased transcription (859 genes) formed the genes of the cuticle and proteolytic processes. Twenty-four hours after the return to Earth, the down-regulated genes did not show significant expression in any functional group.

The combined effect of the transition from the microgravity conditions to the gravity of Earth and from the gas medium of the Foton-M4 satellite to normoxia (third dotted rectangle in Fig 2A, 2B and 2C) resulted in the formation of clusters of up-regulated genes (their numbers were 3LS+12h group vs 3LF group– 1910 and 3LS+24h vs 3LF– 1302) that encode proteins involved in metabolic processes and cuticle formation. The clusters of down-regulated genes (24 hours after the satellite landing, their numbers were 3LS+24h group vs 3LF group– 2702) encoded proteins involved in morphogenesis, transcriptional regulation and were co-localized with the plasma membrane. Twelve hours after the satellite landing, no such clusters were formed.

The exposure to the 12-day space flight (in RS of the ISS), regardless of whether it was a primary action (5LSF group) or secondary action (5LFF group), resulted in the formation of the following gene clusters among the up-regulated and down-regulated genes (fourth dotted rectangle in Fig 2A, 2B and 2C): up-regulated genes (their numbers were 5LSF group vs 5LSS group– 813, 5LFF vs 5LSS– 818, and 5LFF vs 5LSF– 1945) included the metabolic genes involved in cuticle formation and the down-regulated genes (their numbers were 5LSF group vs 5LSS group– 3337, 5LFF vs 5LSS– 6098, and 5LFF vs 5LSF– 3370) included the genes involved in morphogenesis, differentiation, cytoskeletal organization, and transcriptional regulation. The down-regulated genes in the 5LSF group did not show significant expression in any functional group.

### The mRNA content of genes that encode cytoskeletal proteins

It should be noted that changes of transcription level of following estimated genes by qPCR and NGS analysis were in the same direction.

**Fig 3:**
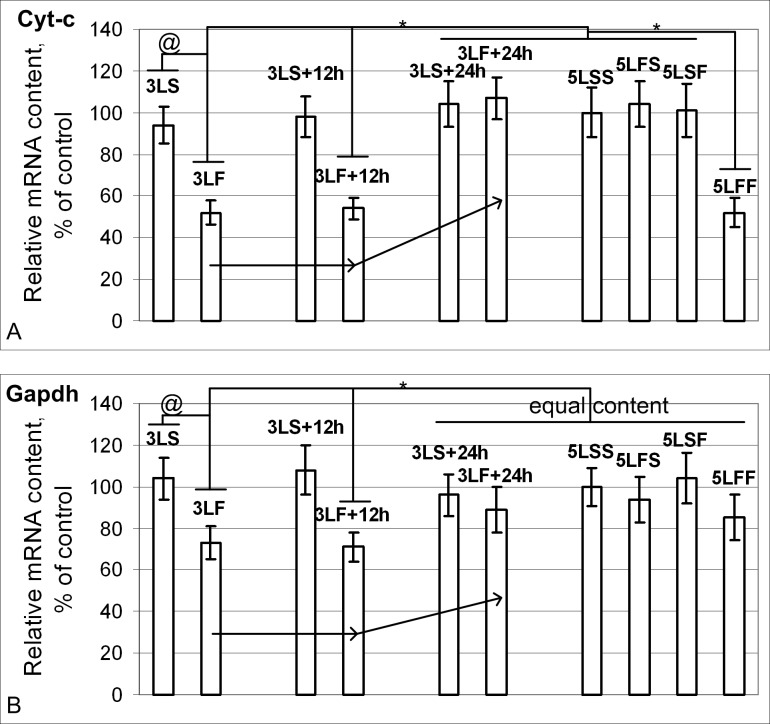
Relative mRNA content of genes (qPCR data) that encode metabolic proteins. (A) The *Cyt-c* mRNA content in 3LS, 3LS+12h, 3LS+24h, 5LSS, 5LFS and 5LSF groups was the same. In the 3LF and 3LF+12h groups, the *Cyt-c* mRNA was reduced by 48% and 46% compared with the 5LSS group (p<0.05) but was restored to the control level in the 3LF+24h group. In the 5LFF group, the *Cyt-c* mRNA content was reduced by 48% compared with the 5LSS group (p<0.05). (B) The *Gapdh* mRNA content in the 3LS, 3LS+12h, 3LS+24h, 3LF+24h, 5LSS, 5LFS, 5LSF and 5LFF groups was the same. In the 3LF and 3LF+12h groups, *Gapdh* mRNA was reduced by 27% and 29% compared with the 5LSS group (p<0.05).

The mRNA contents of genes that encode metabolic proteins such as cytochrome *c Cyt-c* (Fig 3A) and glyceraldehyde-3-phosphate dehydrogenase *Gapdh* (Fig 3B) in the larvae recorded 1.5 and 12 hours after the satellite’s flight were significantly reduced compared with the corresponding control groups but were increasing compared with the control groups 24 hours after landing. The “primary” 12-day flight group did not show changes in the contents of *Gapdh* and *Cyt-c* mRNAs, and the mRNA content of the gene that encodes cytochrome *c* was reduced in the “repeated” flight group.

**Fig 4:**
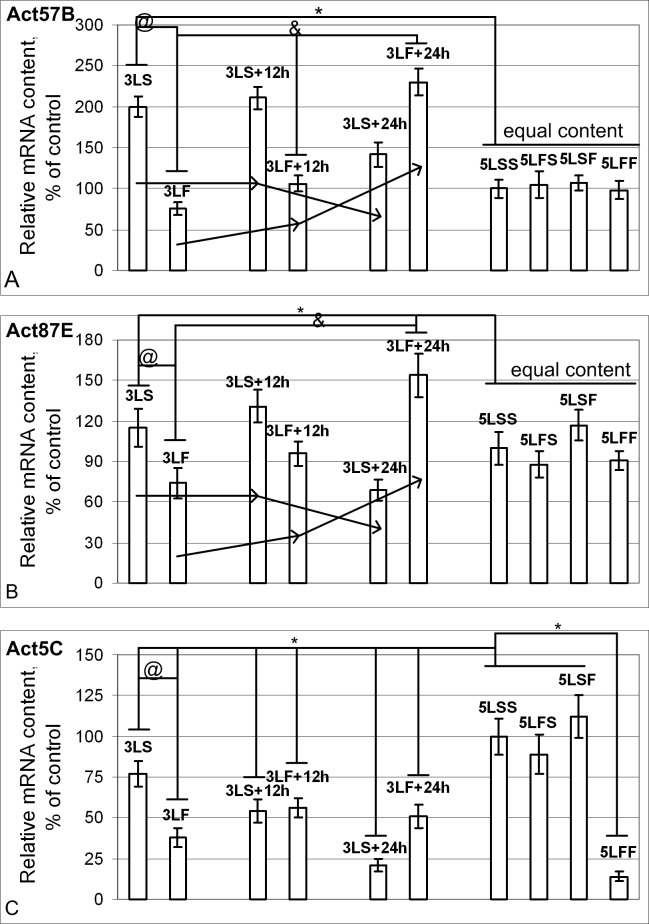
Relative mRNA content of genes (qPCR data) that encode actin isoforms. (A) The *Act57B* mRNA content was increased by 100% in the 3LS group (p<0.05), by 111% in the 3LS+12h group(p<0.05), and by 42% in the 3LS+24h group (p<0.05) compared with the 5LSS group. In 3LF group, *Act57B* mRNA was reduced by 24% (p<0.05), it was the same in the 3LF+12h group, and it exceeded the level of the 5LSS group by 130% in the 3LF+24h group (p<0.05). In the 5LSS, 5LFS, 5LSF, and 5LFF groups, the mRNA content was the same. (B) The *Act87E* mRNA content was the same in the 3LS group, was increased by 31% in the 3LS+12h group (p<0.05), and was decreased by 31% in the 3LS+24h group (p<0.05) compared with the 5LSS group. *Act87E* mRNA was reduced by 26% (p<0.05) in the 3LF group, was the same in the 3LF+12h group, and was increased by 54% in the 3LF+24h group (p<0.05) compared with the 5LSS group. Similar to *Act57B*, the mRNA content was the same in the 5LSS, 5LF, 5LSF, and 5LFF groups. (C) The *Act5C* mRNA content in the 3LS, 3LS+12h, 3LS+24h, 3LF, 3LF+12h and 3LF+24h groups, as well as the 5LFF group was reduced by 23%, 46%, 79%, 62%, 44%, 49% and 86% compared with the 5LSS group (p<0.05).

The contents of Act57B (Fig 4A) and Act87E mRNAs (Fig 4B) increased under hypoxic conditions but were decreased upon return to the normoxic conditions. However, based on the combined effect of hypoxia and space flight factors, microgravity apparently had a decisive role in reducing the levels of *Act57B* and *Act87E* mRNAs in the 3^rd^ generation larvae, and the subsequent influence of the gravity of Earth increased the content. The *Act5C* mRNA content (Fig 4C) was reduced in the synchronous control groups and in the groups after the flight.

**Fig 5:**
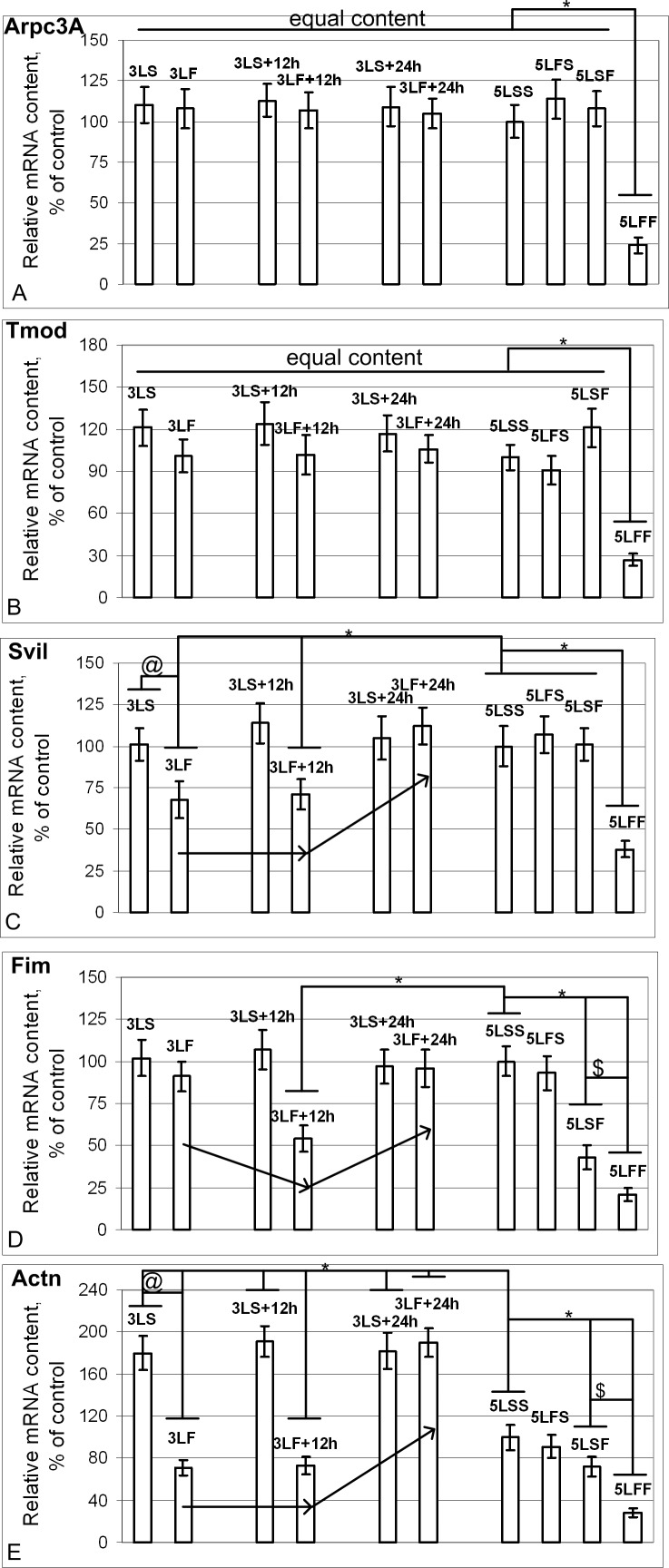
Relative mRNA content of genes (qPCR data) that encode actin-binding proteins. (A) and (B) The *Arpc3A* (A) and *Tmod* (B) mRNA contents were the same in the different groups, with the exception that the contents were reduced by 76% and 73%, respectively, in the 5LFF group compared with the 5LSS group (p<0.05). (C) The *Svil* mRNA content in the 3LS, 3LS+12h, 3LS+24h, 5LSS, 5LFS, and 5LSF groups was the same. The *Svil* mRNA content was significantly reduced by 32% and 29% in the 3LF and 3LF+12h groups, respectively, compared with the 5LSS group (p<0.05), whereas the levels were restored to the control level (5LSS group) in the 3LF+24h group. In the 5LFF group, the *Svil* mRNA content was reduced by 62% compared with the 5LSS group (p<0.05). (D) The *Fim* mRNA content in the 3LS, 3LS+12h, 3LS+24h, 5LSS, 5LFS groups was the same. The *Fim* mRNA content was the same in the 3LF group and 5LSS group, it was decreased by 46% in the 3LF+12h group (p<0.05), and was restored to the control level (5LSS group) in the 3LF+24h group. In the 5LSF group, the *Fim* mRNA content was reduced by 57% compared with the 5LSS group (p<0.05) and by 79% compared with the 5LFF group (p<0.05). (E) The *Actn* mRNA content in the 3LS, 3LS+12h and 3LS+24h groups was increased by 80%, 91% and 82% (p<0.05), respectively, compared with the 5LSS group. The *Actn* mRNA content was significantly reduced by 29% and 27% in the 3LF and 3LF+12h groups, respectively, compared with the 5LSS group (p<0.05) and was increased by 90% in the 3LF+24h group (p<0.05). In the 5LSF and 5LFF groups, *Actn* mRNA content was reduced by 28% and 72% (p<0.05), respectively, compared with the 5LSS group.

The mRNA content of genes that encode proteins that bind actin monomers, *Arpc3A* (Fig 5A) and *Tmod* (Fig 5B), did not change in the 3^rd^ generation larvae recorded at different intervals after the satellite landing. The 5LSF group, i.e., after the 12-day space flight, also showed no changes. However, the re-exposed group showed a significant reduction in the mRNA content of these genes.

The mRNA content of genes that encode proteins that bind actin filaments changed in different ways. The mRNA content of *Svil* (Fig 5C) was only changed in the re-exposure group and was significantly reduced compared with the control group. The mRNA content of *Fim* (Fig 5D) in the group of the larvae recorded 1.5 hours after the satellite landing was the same as the control level; after 12 hours, the *Fim* mRNA levels were sharply decreased, and after 24 hours, they were restored to the control values. After the primary 12-day flight, the mRNA content of *Fim* in the 5LSF group was below the control level and was even lower in the re-exposed 5LFF group. The mRNA content of alpha-actinin *Actn* (Fig 5E) in the synchronous control groups exceeded the level of the 5LSS group during the satellite’s flight, which apparently may be caused by the increased content of actin isoforms under hypoxic conditions. However, similar to actin, the mRNA content of *Actn* in the larvae recorded 1.5 hours and 12 hours after the satellite landing was significantly decreased compared with the corresponding control groups and sharply increased after 24 hours.

**Fig 6:**
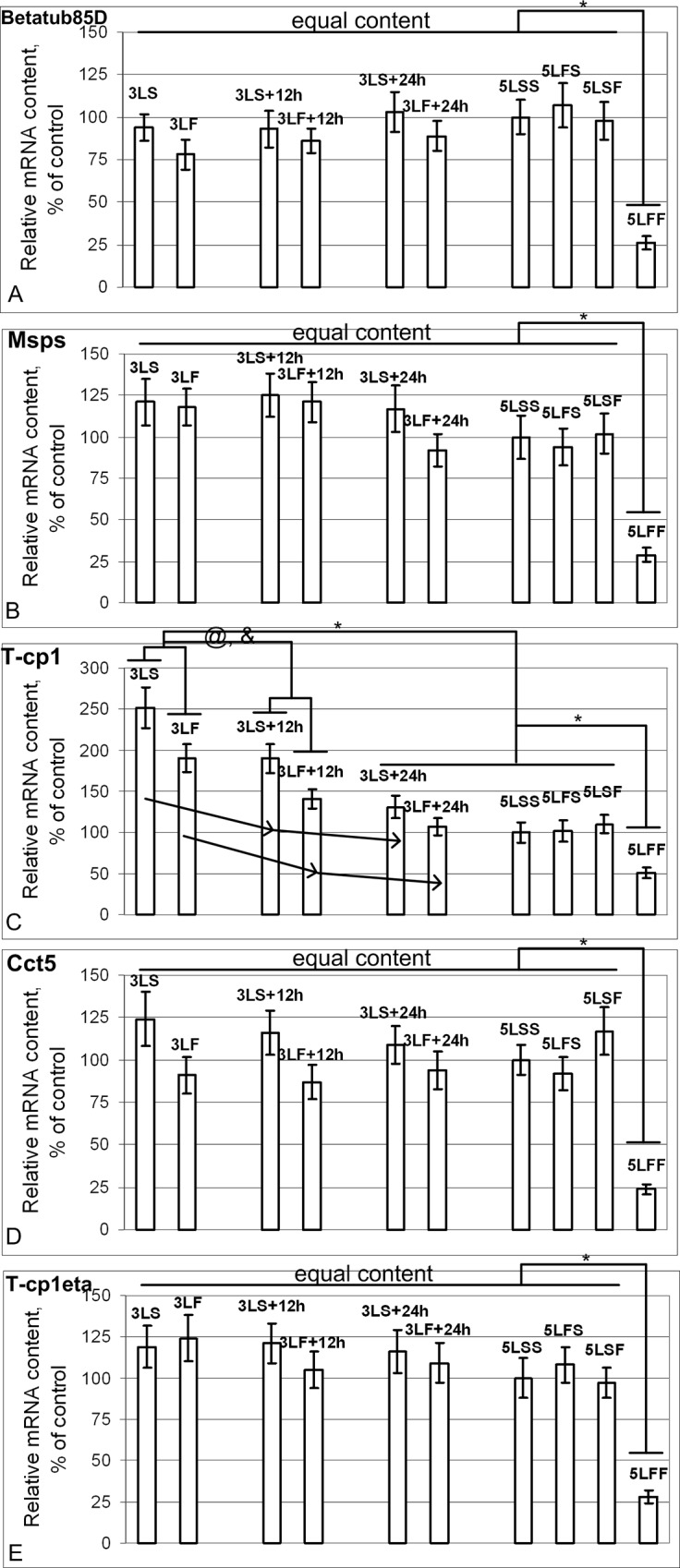
Relative mRNA content of genes (qPCR data) that encode proteins of microtubule cytoskeleton. (A), (B), (D), (E) The *Betatub85D* (A), *Msps* (B), *Cct5* (D) and *T-cp1eta* (E) mRNA contents were the same in all study groups, with the exception of the 74%, 71%, 76% and 72% decreases in the 5LFF group compared with the 5LSS group (p<0.05), respectively. (C) The *T-cp1* mRNA content in the 3LS and 3LS+12h groups exceeded the level of the 5LSS group by 152% and 90% (p<0.05), respectively, and by 91% and 41% (p<0.05) in the 3LF and 3LF+12h groups, respectively. The *T-cp1* mRNA content was the same as the control level in the 3LS+24h, 3LF+24h, and 5LSF groups. The *T-cp1* mRNA content in the 5LFF group was reduced by 49% compared with the 5LSS group (p<0.05).

The mRNA content of genes that encode the tubulin cytoskeleton components *Betatub85D* (Fig 6A), *Msps* (Fig 6B), *Cct5* (Fig 6D) and *T-cp1eta* (Fig 6E) did not change in any of the groups, except for the re-exposed 5LFF group in which the mRNA content was significantly reduced. However, the mRNA content of the gene that encodes one of the tubulin cytoskeleton components involved in protein folding, *T-cp1* (Fig 6C), greatly exceeded the control level 1.5 hours after the end of the experiment, decreased after 12 hours, and was at the control level after 24 hours. Similar dynamics were observed in the groups after the flight, but the mRNA contents in the corresponding post-flight groups were reduced compared with the synchronous control groups.

### Protein content in the membrane and cytoplasmic fractions

**Fig 7:**
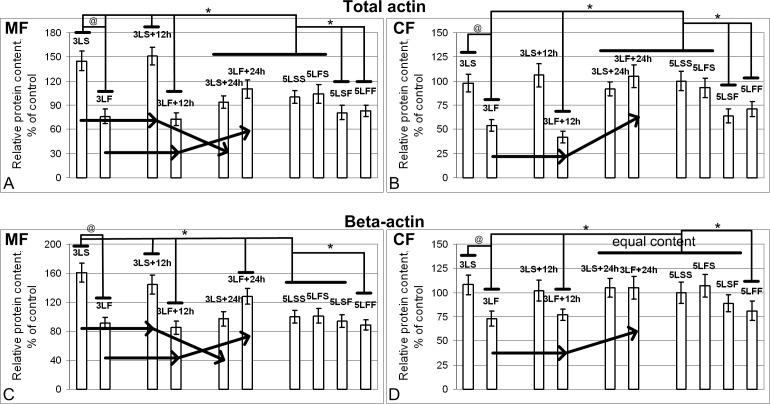
Relative content of actin in the membrane (MF) and cytoplasmic (CF) fractions. (A) and (B) Total actin. The total actin content in the MF from the 3LS and 3LS+12h groups exceeded the level in the MF from the 5LSS group by 45% and 51% (p<0.05) but was not significantly different in the CF. In the 3LS, 3LS+12h, 5LSF, and 5LFF groups, the total actin contents in the MF and CF were reduced by 24% and 46% (p<0.05), 27% and 58% (p<0.05), 19% and 36% (p<0.05), and 17% and 29% (p<0.05), respectively, compared with the 5LSS group. (C) and (D) Beta-actin. The beta-actin content in the MF from the 3LS and 3LS+12h groups exceeded the level of 5LSS group by 61% and 44% (p<0.05), respectively, but remained unchanged in the CF. The beta-actin content in the CF from the 3LF and 3LF+12h groups was reduced by 27% and 29% (p<0.05) compared with the CF of the 5LSS group but remained unchanged in the MF. In the 3LF+24h group, the beta-actin content in the MF exceeded the level in the MF of the 5LSS group by 28% (p<0.05) but remained unchanged in the CF.

The total actin content in the membrane fraction (Fig 7A) increased under hypoxic conditions (in the synchronous control groups during the satellite’s flight) and decreased to the control level after a 24-hour exposure to the normoxic conditions. The actin content (Fig 7B) was reduced after the satellite’s space flight (i.e., the combined action of hypoxia and microgravity) and the 12-day space flight, and the reduction was even more significant in the cytoplasmic fraction.

The changes in the beta-actin content were the same, but less protein was expressed (Fig 7C and 7D). Thus, hypoxia increased the beta-actin content in the membrane fraction (3LS and 3LS+12h), and its combined action with the microgravity decreased the content in the cytoplasmic fraction (3LF and 3LF+12h). It should be noted that the 12-day space flight did not lead to significant changes in the beta-actin content either during the primary exposure or during the “repeated” exposure.

**Fig 8:**
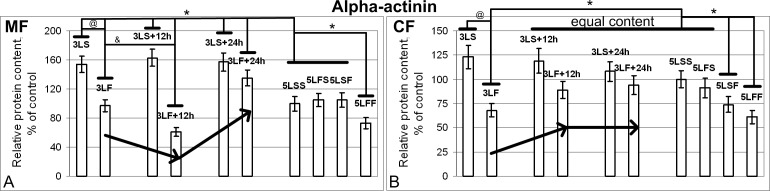
Relative content of alpha-actinin in the membrane (MF) and cytoplasmic (CF) fractions. (A) membrane fraction (MF). (B) cytoplasmic fraction (CF). The alpha-actinin content in the MF of the 3LS, 3LS+12h and 3LS+24h groups was higher than the MF of the 5LSS group by 54%, 63% and 57% (p<0.05), respectively, but remained unchanged in the CF. The alpha-actinin content in the MF of the 3LF group was the same as the MF of the 5LSS group but was reduced by 32% in the CF (p<0.05). The content in the MF of the 3LF+12h group was reduced by 32% (p<0.05), and in the CF, it was restored to the CF level of the 5LSS group. Then, the alpha-actinin content in the MF of the 3LF+24h group was increased by 35% compared with the MF of the 5LSS group (p<0.05), but it remained at the same level in the CF. The alpha-actinin content in the MF of the 5LSF group was the same as the MF of the 5LSS group, but in the CF, it was decreased by 26% (p<0.05). In the MF and CF of the 5LFF group, the alpha-actinin content was reduced by 27% and 39% compared with the MF and CF of the 5LSS group (p<0.05), respectively.

Similar to the total actin and beta-actin contents, the alpha-actinin content increased in the membrane fraction (Fig 8A) but did not increase in the cytoplasmic fraction (Fig 8B) under the hypoxic conditions. Twenty-four hours after the exposure to normoxic conditions, the alpha-actinin level was still significantly higher than the control level (3LS, 3LS+12h, and 3LS+24h groups). However, 1.5 hours after the satellite landed (3LF group), the alpha-actinin content in the membrane fraction did not differ from the control level (significantly decreased compared with the 3LS group of the corresponding synchronous control), but it was reduced by 32% in the cytoplasmic fraction (p<0.05). Furthermore, 12 hours after the satellite landed, the content in the membrane fraction decreased significantly and increased greatly in the cytoplasmic fraction, reaching the control level. Then, 24 hours after exposure to the gravity of Earth, the alpha-actinin content in the membrane fraction significantly exceeded the control level but remained unchanged in the cytoplasmic fraction. The 12-day flight of the primary exposure 5LSF group resulted in reduced alpha-actinin content, similar to the 3LS group, but only in the cytoplasmic fraction. The “repeated” exposure (5LFF group) led to the reduced alpha-actinin content in both fractions, although the reduction in the cytoplasmic fraction was more obvious.

**Fig 9:**
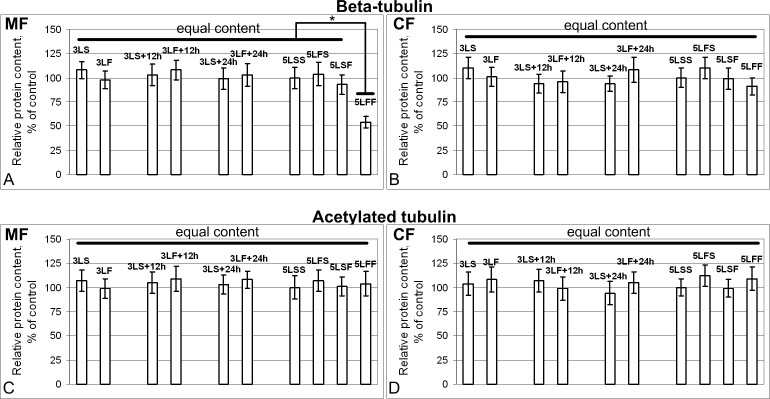
Relative content of tubulin in the membrane (MF) and cytoplasmic (CF) fractions. (A) and (B) Beta-tubulin. In all study groups, the beta-tubulin content remained at the same level in both the MF and CF, with the exception of the 46% decrease in the MF of the 5LFF group (p<0.05) compared with the MF of the 5LSS group. (C) and (D) Acetylated tubulin. The acetylated tubulin content remained the same in both fractions from all of the groups.

The change in the beta-tubulin content (Fig 9A and 9B) was only observed in the membrane fraction of larvae proteins obtained after the “repeated” exposure under the 12-day space flight conditions. The content of acetylated tubulin did not change (Fig 9C and 9D) in any of the study groups.

## Discussion

During the experiment, we succeeded, for the first time, in creating the third generation of the highly organized multicellular eukaryotic organism *Drosophila melanogaster* under weightless conditions, created the fourth generation on Earth, and arranged another space flight and created the fifth generation larvae (relative to the start of the experiment) in near-Earth orbit from the third stage of the *Drosophila*.

The main purpose of the research was molecular screening of the obtained organisms targeted to the study of cytoskeletal proteins. Despite of the main purpose we would like to note that we did not observe any morphological changes in larvae after the space flights.

The first steps in the screening analysis of the fruit fly transcriptome were implemented by Arbeitman et al. [19], who studied the differential expression of one-third of the genome during development. The authors identified the new genes involved in a wide range of processes, including early embryo structuring and the adult aging process [19]. Then, the data from the transcriptome analysis of 30 stages of fruit fly development were provided [20]. Furthermore, new genes, transcripts, and proteins from cultured cell lines of *Drosophila melanogaster*, dissected organ systems, and environmental perturbations were identified [21].

The genome-wide transcriptional profile showed that the presence of the reduced gravity level during *Drosophila* metamorphosis in the experiment at the ISS resulted in changes in the gene expression profile, primarily in stress reactions [22], which was confirmed in the experiments conducted using the random position machine [23]. The products of these differentially expressed genes are involved in respiration, development, morphogenesis processes, and the formation of the stress response [24].

Our results show that the transition from hypoxia to normoxia, as well as the exposure to weightless conditions, increased the transcription of metabolic genes and cuticle components and decreased the transcription of genes involved in morphogenesis, cell differentiation, cytoskeletal organization and genes associated with the plasma membrane. “Repeated” exposure to microgravity for 12 days resulted in an even more significant increase/decrease in the transcription of the same genes.

In contrast, the transition from the microgravity conditions to the gravity of Earth increased the transcription of genes encoding proteins involved in morphogenesis, cytoskeletal organization, cell motility and transcriptional regulation and decreased the transcription of cuticle genes and genes involved in proteolytic processes.

### Influence of the gas composition of the medium

By comparing the 5LSS control group with the 3LS group of synchronous controls during the satellite’s flight, we can suggest that the slight hypoxia observed during the flight increased the expression of genes that encode one of the beta-actin isoforms (*Act57B*) and one of the actin-binding proteins (*Actn*) among all the genes studied here. In addition, the expression of one of the tubulin-binding proteins, the chaperonin *T-cp1*, also increased. It should be noted that the slight change in the gas composition of the medium did not lead to changes in the expression of metabolic genes (*Cyt-c* and *Gapdh*).

In contrast, after the space flight with the same conditions of the gas medium (3LF group vs 3LS and 5LSS groups), the expression of the same cytoskeletal genes and the metabolic genes decreased. However, the expression of chaperonin *T-cp1* also increased under these conditions.

Thus, the control groups allowed us to differentiate the effects of slight hypoxia and microgravity: hypoxia increases the expression of the actin cytoskeleton genes without affecting the tubulin cytoskeleton genes, and microgravity decreases the expression of these genes.

### Transition from a long space flight to Earth’s gravity

In general, the return to Earth and normoxic conditions after 12–24 hours resulted in the restoration of the expression of the studied genes and the protein contents to the control level.

However, we think that the dynamics of the changes in the expression levels of one of the actin-binding proteins, fimbrin (*Fim*), are the most essential changes. The gas composition of the medium did not affect the gene expression (in all control groups, the mRNA content of this gene remained unchanged). Furthermore, after the space flight (in the 3LF group), the mRNA content also remained unchanged. However, 12 hours after the satellite landed (i.e., upon increased external mechanical stress), the mRNA content of the gene decreased compared with the level immediately after landing and the control, and after 24 hours, *Fim* expression was restored to the control level.

Our results corroborate our previous results obtained from mice in model experiments and after the space flight of the Bion-M1 biological satellite (2013, Russia); the increase in the external mechanical stress (gravity) decreases the alpha-actinin-1 content (leading to the reduced content of another isoform, alpha-actinin-4) in the membrane fraction of proteins and an increase in the cytoplasmic fraction after 6 hours, subsequent (after 12 hours) to the reduced expression of the corresponding gene level [25,26,27,28,29]. These results allowed us to assume that different deformations of the cortical cytoskeleton are the primary mechanical sensor of the increase and decrease in the external mechanical stress, resulting in the separation of various actin-binding proteins and, consequently, the triggering of different signalling pathways [30].

However, the principle of the primary reception mechanism of the external mechanical tension can apply to different cells, including distantly related organisms. It should be noted that we did not observe changes in the fimbrin gene expression upon changes in the external mechanical conditions in muscle cells of mice [29]. However, because *Drosophila* has no alpha-actinin-4 isoform, its role in the cell may be fulfilled by fimbrin, for which its localization and probably one of the functions were assigned to alpha-actinin-4 during evolution. Of course, this assumption is a hypothesis that requires additional experiments, primarily under space flight conditions.

### Effects of long, short and “repeated” flight

The 3LF group includes the 3^rd^ generation larvae that were created in space (i.e., from germ cells formed in space), whereas the 5LSF group includes, in fact, the first generation created in space (i.e., from germ cells formed on Earth). The 3LF group features the reduced expression of metabolic genes and genes that encode proteins of the actin cytoskeleton (except for fimbrin). The 5LSF group did not show these changes, except for the similar decrease in the expression of alpha-actinin and, consequently, the decrease in the expression of fimbrin. The expression of genes that encode proteins that form microtubules remained unchanged in both groups (except for the chaperonin *T-cp1* in the 3LF group, which was caused by the changes in the gas composition).

Moreover, the 5LFS group includes the 1^st^ generation larvae created on Earth from germ cells formed in space; the 5LFF group includes the first generation created in space from germ cells formed in space that were developed on Earth (in fact, it is the fifth generation relative to the start of the experiment). The 5LFS group showed very little difference in the cytoskeletal gene expression pattern, whereas the 5LFF group showed significantly decreased expression of genes that encode actin proteins and even the tubulin cytoskeleton.

The significant difference in the expression patterns of larval genes of the actin cytoskeleton that emerged as a result of the merger of germ cells formed in space and the larvae that emerged from the germ cells formed on Earth suggests that gravity represents one of the epigenetic factors for which the mechanism of action still remains unclear.

However, the actin content, especially in the membrane fraction of proteins, despite the sharp decrease in the level of expression in flight groups did not fall below 60%, which probably allowed the cortical cytoskeleton to exercise its functions related to the maintenance of the cell shape and the formation of the contractile ring. Furthermore, the content of the acetylated tubulin which represents a microtubule stability marker remained unchanged in all study groups (including the group of the second flight), which, respectively, enabled to form the cleavage spindle and create subsequent generations.

## Conclusions

The obtained results show that it is possible to obtain third to fifth generations of a highly organized multicellular Earth organism under changed gravity conditions (in the cycle “weightlessness–Earth–weightlessness”), which preserves fertility. However, a number of changes in the level of expression and content of cytoskeletal proteins, which are the key components of the spindle apparatus and the contractile ring of cells, were observed. The preservation of fertility implies that human exploration of other planets (such as Mars) and outer space, with alternating stays under weightless conditions and different gravities, may be possible in terms of species preservation.
